# Toward a more comprehensive autism assessment: the survey of autistic strengths, skills, and interests

**DOI:** 10.3389/fpsyt.2023.1264516

**Published:** 2023-10-06

**Authors:** Sara Eileen O'Neil Woods, Annette Estes

**Affiliations:** ^1^Autism Center, University of Washington, Seattle, WA, United States; ^2^Institute on Human Development and Disability, University of Washington, Seattle, WA, United States; ^3^Discover Psychology Services, Lacey, WA, United States; ^4^Department of Speech and Hearing Sciences, University of Washington, Seattle, WA, United States

**Keywords:** autistic, strengths, autism diagnosis, stigma, neurodiversity, autism assessment, autism

## Introduction

Autism is primarily defined by its deficits in the Diagnostic and Statistical Manual of Mental Disorders (DSM-5) (1). However, autism can be defined as a natural, valuable part of human experience in which the ability to thrive depends on the match between the individual and their social context (2–8). Autistic strengths have been noted since autism was first defined (9). A growing body of literature has demonstrated how strengths in social communication, focused interests, stimming, sensory abilities, systems thinking, and cognition can be part of autism (10–13). Despite the neurodiversity movement, autism is still associated with stigma (14–16). Diagnostic evaluations often focus exclusively on problems without considering strengths (17, 18). Most questionnaires, observational tools, and interview questions tabulate problems to determine if someone is autistic, but they miss the comprehensive view of what it means to be autistic. The autism diagnostic evaluation is a critical time in a person's life, with some individuals referring to it as the most important experience of their life (19, 20). When an individual is first discovering they or their child is autistic, providing strengths-based information can provide an alternative to some of the stigmatizing messages they may have heard about autism. This can be shared in addition to a discussion about some of the anticipated challenges (21, 22). Autism-specific strengths-based measures that allow clinicians to assess for autistic strengths during diagnosis are needed. The Survey of Autistic Strengths, Skills, and Interests (SASSI, presented in the Supplementary Table 1) is a set of questions that can be integrated into the clinical interview with an adult or caregiver to explore and identify common autistic strengths. It is meant to be used along with a comprehensive battery that also includes challenges.

## Social communication strengths

By definition, autistic people fail to conform to social norms, but a deficit-based diagnostic process can overshadow the value of nonconformity (16). Studies have shown that non-autistic people are more likely to conform to the majority by choosing an incorrect response if they think it is popular whereas autistic people tend to choose the correct answer even if that answer is unpopular. This autistic willingness to go against the crowd when correct has been demonstrated in autistic children (23) and adults (24). This research is based on small sample sizes, but many autistic activists cite their autism as giving them the strength to speak out (25). Non-autistic people tend to change their prosocial behavior (e.g., giving to charity) depending on whether they are being watched, whereas autistic people behave more consistently across contexts (26). In one study, autistic people were more likely to refuse to make an immoral choice (giving to a “bad charity”) even if it benefitted them (gaining money). Non-autistic people changed their behavior depending on whether they were being watched, but autistic people were consistent (27). Many autistic people also identify honesty as an autistic strength (28, 29). Autistic people may be characterized as “lacking a filter,” but being honest and direct saves time and allows for clearer understanding. Learning to express oneself directly can be an important intervention for neurotypical people (30). Autistic people point out that being guided by their own internal ethical compass and being less influenced by what other people think is a strength (31). However, autism stigma has led even this moral consistency to be conceptualized as a deficit [for an example, see (27)].

Need for solitude was one of the first characteristics identified as defining autism (32). This has been framed as a deficit, but enjoyment of solitude is also a strength. It is associated with lower levels of depression and anxiety (33). Experience sampling studies suggest that autistic people tend to enjoy solitude and may not feel lonely when alone (34). However, autistic people often highly value friendships as well. Making friends is a major developmental achievement of middle childhood (35). Friendship can protect against depression and anxiety (36). Some autistic people are selective in their friendships. They may keep their circles small (37, 38) and value friendships in which they can be authentic (38, 39). Autistic people often connect well across age groups (40–43). Research on the double empathy problem (44, 45), has shown that autistic people can often connect with other autistic people more effectively than non-autistic people can (46). Autistic pairs tend to have stronger rapport than mixed-neurotype pairs (47). Autistic people report experiencing relationships with other autistic people as highly satisfying and less tiring (48, 49). Autistic people also have a strong ability to connect with other autistic people online (50–52). Assessing a client's autistic friendships and online social network could inform ways to reduce the isolation autistic people sometimes experience (53), inform our understanding of social support networks, and identify support needed to further achieve satisfying relationships.

### Focused interests and stimming

Focused interests can offer a sense of wellbeing (54–56), facilitate social connection (57), guide employment opportunities (58, 59), and strengthen academic skills and executive functioning (57, 60–62). Although the DSM-5 views “restricted interests” as deficits (1), clinicians can take a more wholistic view by identifying clients' interests and considering the potential opportunities they offer. Because autistic people tend to experience monotropism (63), they may be able to devote long periods of time to studying or talking about one subject, which often leads to expertise and mastery (64, 65). Some autistic people have animals as a special interest and many describe themselves as connecting well with animals (29). Interests can facilitate friendships, provide educational and vocational focus, as well as being pleasurable, so should be assessed directly.

Autistic people engage in repetitive movements primarily as a way to cope with intense thoughts or sensory experiences (66), but these are framed as deficits in part because engaging in unusual behavior is stigmatized (66, 67). Although some repetitive motor movements can indicate neurological problems (68, 69) or contribute to back pain or self-injury (70), they can also provide pleasure and serve a regulatory purpose. If clinicians can be mindful of our biases and ask about stimming from a positive perspective, we may help guide our clients to see the ways in which stimming helps them.

### Sensory strengths

Autistic people often have sensory sensitivities, which may cause distress in certain environments. This, along with our profession's predisposition to view autism through a deficit-lens, has resulted in sensory sensitivities being framed as deficits on most questionnaires. This has limited research into whether sensory differences may also be strengths. Some previous studies have suggested that autistic people tend to perform more poorly on certain sensory tasks, such as identifying individual smells (71) and switching their attention between different sounds (72). Despite the challenges posed by sensory sensitivities, research has also reported autistic strengths, such as increased likelihood of perfect pitch (73), ability to identify smells that are mixed together (74), ability to recognize sounds that are mixed together (75), and performance on visual search tasks (76, 77). Attention to detail is one of the most prominent self-reported autistic strengths (29). These findings have led some researchers to refer to autistic people as perceptual experts (76). Sensory sensitivity itself may be linked to increased capacity. For example, autistic people who are more sensitive to sounds may perform better on tests of auditory capacity [ability to detect specific sounds mixed with multiple distracting sounds; (78)]. Sensory differences may also inform creative pursuits and coping strategies.

Asking clients about their positive sensory experiences can offer guidance in empowering clients to select and modify environments to support autistic flourishing. Most existing questionnaires and interview questions assess whether autistic people are bothered by sensory input, but very few tools assess sensory strengths. The Monteiro Interview Guidelines for Diagnosing the Autism Spectrum, Second Edition [MIGDAS-2, (79)] does assess sensory experiences that the individual enjoys and attention to detail. The SASSI offers additional assessment questions.

### Systems and routine

From a young age, many autistic children line-up toys, gaze at them from different angles, and arrange them by color or shape. Some are drawn to pre-existing systems like alphabetical and numerical order (80). This has been framed as nonfunctional (1) and as obstructing or distracting from more productive types of play. A frequently cited paper introduces restricted interests and repetitive behaviors as constituting “a major barrier to learning and social adaptation” (81). Recent research has shown, however, that engaging in this autistic way of playing at preschool age is linked to improved nonverbal reasoning skills at school-age (82). In addition to bringing joy to autistic children, which in and of itself is a worthy goal (83, 84), this interest in systems means that many autistic people are good at creating their own systems for making their environments work (28). Many autistic people also thrive on routine (85), which can be an advantage in many environments.

### Cognitive strengths

There is no one autistic profile when it comes to cognition, but there are certain strengths that seem to be associated with autism across the lifespan, and may appear even among autistic children classified as having intellectual disabilities or as being untestable (86, 87). Autistic people often outperform non-autistic people on tasks assessing visual-spatial reasoning [e.g., Block Design on the Wechsler tests, (88)], other nonverbal tasks that required identifying visual features embedded among other distracters [e.g., visual search tasks, (77, 89), and embedded figures tests, (86, 87)], and certain executive functioning tasks (90). Recent longitudinal research has shown that early performance on some of these specific types of tasks (e.g., embedded figures tasks) is linked to non-verbal intelligence as autistic children get older (82).

## Discussion

Clearly autism is more than the set of deficits we have traditionally been taught to assess and evaluate. To move toward overcoming the stigma that permeates our diagnostic assessments we must expand and clean the lenses through which we view autism. Asking directly about autistic strengths can help us see our clients' experiences more clearly and make more effective recommendations to help them move forward while embracing who they already are. The SASSI is a newly developed tool meant to inspire future research on assessing autistic strengths. Future research could include focus groups or interviews with autistic adults to further refine the items, pilot studies with small groups of clinicians who can apply it to children and adults, and exploration of how it might be modified to include Likert scales. Our hope is that the SASSI can serve as a step toward inspiring future research and refining our diagnostic evaluations as we recognize together the value of expanding our conceptualization of autism.

## Author contributions

SW: Conceptualization, Writing—original draft, Writing—review and editing. AE: Supervision, Writing—original draft, Writing—review and editing.
