# Supplementary material for: Toward a more comprehensive autism assessment: the survey of autistic strengths, skills, and interests
Source: Front Psychiatry. 2023 Oct 6;14:1264516. doi: 10.3389/fpsyt.2023.1264516 (PMC10587489; doi:10.3389/fpsyt.2023.1264516)
Supplement: Supplementary file 1 [file Table_1.docx]

**Table 1. Survey of Autistic Strengths, Skills, and Interests – Adult Version**

Note: These questions are meant to be asked in interview format as part of a larger clinical interview. Questions are aimed at identifying strengths commonly associated with autism (but not necessarily exclusive to autism). Responses to the questions are not diagnostic of autism on their own.

**_________________________________________________________________________________**

Social Non-Conformity and Justice

1. Do you have a strong sense of justice?
2. Can you describe times where you have been willing to go against the majority because of something you believed in or because of something that was important to you?

Solitude

1. Are you able to spend long periods of time alone doing something you enjoy? Do you appreciate solitude? Do you enjoy your own company? Do any examples come to mind?

Honesty/Direct Communication

1. How direct and straightforward do you think you are? Are you generally honest? Do you tend to ‘beat around the bush’ or are you willing to ‘tell it like it is’? Can you give some examples?

Connecting with Others

1. Do you connect well with autistic people? People who describe themselves as “neurodivergent”? People with disabilities?
2. How are you at picking up on certain subtle cues that some other people might miss? Do examples come to mind?

Friends

1. How selective are you when it comes to friendships? Do you have specific requirements of friends? Can you explain?
2. How do you do with connecting with other people online? Can you give examples?
3. How important is it for you to tell people directly if their behavior is wrong or if it upsets you?
4. How willing are you to be friends with people from different age groups?
5. Do you have an inner circle or close group of people that you can trust?

Interests

1. How passionate are you about your interests? Are you able to focus on them for extended periods of time? Do you feel energized by them? Do examples come to mind?
2. Do you find yourself able to get very interested in details about particular topics?
3. Have your focused interests helped you in school or work?
4. Have they helped you connect with other people?

Systems/Routine

1. How are you at organizing materials?
2. To what extent do you thrive on routine?
3. How are you at creating routines or systems for yourself? For other people?
4. How do you do if other people create routines for you?
5. Are you okay with following a similar schedule repeatedly? Are you less likely to get bored by routine than other people, do you think?

Physical Movement

1. What are some of your unique ways of calming yourself or dealing with tension?

Sensory

1. Do you think that you experience heightened awareness of certain aspects of sensory stimuli (e.g., sights, sounds, smells, tastes, textures)?
2. Do you appreciate certain sensory experiences in a way that other people cannot?
3. Do you have a unique way of experiencing the world that helps you in your creative, musical, or artistic activities? Can you think of examples?
4. Did you ever think you might have perfect pitch?
5. What types of sensory experiences bring you joy?

Cognition

1. How are you at detecting patterns?
2. How are you at figuring out how things work? Fixing things?
3. Have you noticed anything interesting about your memory?
4. Did you start reading at an early age?
5. How well do you do with categorizing and organizing information?
6. Do you notice details that other people miss?
7. Have people commented on any unique cognitive skills that you have?

Connecting with Animals

1. How would you describe your ability to connect with animals?

Other

1. What other talents or skills do you have?
2. Do you have any other special abilities, talents, or strengths that you associate with autism?

_____________________________________________________________________

**Table 2. Survey of Autistic Strengths Skills and Interests – Child Version**

Note: These questions are meant to be asked in interview format as part of a larger clinical interview. Questions are aimed at identifying strengths commonly associated with autism (but not necessarily exclusive to autism). Responses to the questions are not diagnostic of autism on their own.

_________________________________________________________________________________

Social Non-Conformity and Justice

1. Does your child have a strong sense of justice?
2. Can you describe times where your child has been willing to go against the majority because of something your child believed in or because of something that was important to them?

Solitude

1. Is your child able to spend long periods of time alone doing something they enjoy? Does your child appreciate solitude? Does your child enjoy their own company? Do any examples come to mind?

Honesty/Direct Communication

1. How direct and straightforward is your child? Is your child generally honest? Do they tend to beat around the bush or are they willing to ‘tell it like it is’? Can you give some examples?

Connecting with Others

1. Does your child connect well with autistic people? People who describe themselves as “neurodivergent”? People with disabilities?
2. How is your child at picking up on certain subtle cues that some other people might miss? Do examples come to mind?

Friends

1. How selective is your child when it comes to friendships? Does your child have specific requirements of friends? Can you explain?
2. How does your child do with connecting with other people online (if allowed)? Can you give examples?
3. How likely is it for your child to speak up if they are bothered by someone’s behavior?
4. How likely is your child to be friends with people from different age groups?
5. Does your child have friends with disabilities? Friends who are neurodivergent?
6. Does your child have a close-knit group of people that they can trust?

Interests

1. How passionate is your child about their interests? Is your child able to remain focused on them for extended periods of time? Does your child seem energized by them? Do examples come to mind?
2. Does your child seem to get very interested in details about particular topics?
3. Have your child’s focused interests helped them in school or work?
4. Have they helped them connect with other people?

Systems/Routine

1. Describe your child’s ability to organize materials.
2. To what extent does your child thrive on routine?
3. How is your child at creating routines or systems for themself? For other people?
4. Is your child able to follow a similar schedule repeatedly? Is your child less likely to get bored by routine than other people, do you think?

Physical Movement

1. What are some of your child’s unique ways of calming themself or dealing with tension?

Sensory

1. Do you think that your child experiences heightened awareness of certain aspects of sensory stimuli (e.g., sights, sounds, smells, tastes, textures)?
2. Does your child seem to appreciate certain sensory experiences in a way that other people cannot?
3. What types of sensory experiences seem to bring your child joy?
4. Does your child have a unique way of experiencing the world that helps in creative, musical, or artistic activities? Can you think of examples?
5. Did you ever think your child might have perfect pitch?

Play

1. Have you noticed anything interesting about your child’s play?
2. Does your children like to line up toys or arrange them in specific ways?
3. Have you noticed anything interesting about your child’s imagination?
4. What about how he or she explores toys and objects?
5. Does your child like to examine objects from different angles?
6. Can your child play well by themself?

Cognition

1. How is your child at detecting patterns?
2. Have you noticed anything interesting about your child’s memory?
3. What talents or skills come more easily to your child than for other people?
4. Did your child start reading at an early age?
5. Can your child read quickly?
6. Does your child notice details that other people miss?
7. How is your child at figuring out how things work or fixing things?
8. Have people commented on any unique cognitive skills that your child has?

Connecting with Animals

1. How would you describe your child’s ability to connect with animals?

Other

1. What other talents or skills does your child have?
2. Does your child have any other special abilities, talents, or strengths that you associate with autism?

___________________________________________________________________________
